# Toxicological Evaluation and Hepatoprotective Efficacy of Rosmarinic Acid-Rich Extract from *Ocimum basilicum* L.

**DOI:** 10.1155/2021/6676998

**Published:** 2021-01-31

**Authors:** Ilham Touiss, Sabir Ouahhoud, Mohamed Harnafi, Saloua Khatib, Oussama Bekkouch, Souliman Amrani, Hicham Harnafi

**Affiliations:** Laboratory of Bioresources, Biotechnologies, Ethnopharmacology and Health, Faculty of Sciences, University Mohammed I, 60000 Oujda, Morocco

## Abstract

Exposure to carbon tetrachloride (CCl_4_) induces acute and chronic liver injuries as well as oxidative stress in rats. The present study was designed to evaluate the *in vivo* toxicity of rosmarinic acid-rich extract from *Ocimum basilicum* (RAE). The acute and subchronic oral toxicity of RAE was evaluated in *Albinos* mice. Hepatotoxicity was induced by the administration of CCl_4_-induced hepatic injury in rats. The hepatoprotective effect of RAE on aspartate aminotransferase, alanine transaminase, alkaline phosphatase, lactate dehydrogenase, bilirubin, total protein, albumin, triglycerides, total cholesterol, low-density lipoprotein, high-density lipoprotein, plasmatic glucose, urea, creatinine, and malondialdehyde was determined in CCl_4_-intoxicated rat. The extract did not produce treatment-related signs of toxicity or mortality in any of the animals tested during acute as well as subchronic toxicity studies. The administration of CCl_4_ resulted in marked increase in plasma hepatic enzymes (*p* < 0.001) and significant decrease of total protein (*p* < 0.001) and albumin (*p* < 0.001) when compared to normal. The RAE at 200 mg/kg body weight lowered significantly (*p* < 0.001) plasma enzyme activities of liver, which is designation of hepatoprotective action of extract. The phenolic extract exerts a significant increase in total protein (*p* < 0.001), and albumin (*p* < 0.001), accompanied with a marked reduction in the levels of malondialdehyde (*p* < 0.001), as compared to CCl_4_-treated group. Our study suggests that RAE may be used as a hepatoprotective agent against toxic effects caused by CCl_4_ and other chemical agents in the liver.

## 1. Introduction

The liver plays a central and important role in regulating various physiochemical functions of the body as digestion, biosynthesis, secretion, and detoxification [1]; it is always vulnerable to different toxic molecules of foreign origin due to its location in the human body [2]. Carbon tetrachloride (CCl_4_) is largely used for experimental induction of liver damage; it is a typical liver toxicant [3]. The main causes of CCl_4_-induced hepatic damage are raised lipid peroxidation and decreased activities of antioxidant enzymes and generation of free radicals [4]. Hepatotoxicity is the term used to describe the functional and structural damage of liver caused by the abuse or misuse of potent medicines and consumption of other hepatotoxic agents; among these agents are alcohol, infections, and chemicals like carbon tetrachloride [5]. However, excessive intake of these chemicals may still result in oxidative damage to tissue organs by massive production of free radicals, which leads to structural and functional damage to the membrane and eventually causing serious toxicity to hepatocytes [6]. Moreover, oxidative stress, which is the result of an excess of reactive oxygen species over the antioxidant defenses of the organism, has been considered as a conjoint pathological mechanism and it contributes to initiation and progression of liver injury [7]. In fact, effects of antioxidants or free radical scavengers have been extensively tested for the prevention and treatment of acute and chronic liver injuries [8]. This is the reason why in several studies antioxidants have shown beneficial effects, particularly for prevention and treatment of liver injury [9, 10]. On this basis, it has been theorized that aggressive antioxidant therapy would improve outcomes in liver damages. On top of that, treatment of liver disease with synthetic drugs may be inadequate or have serious adverse effects [11]. That is why it is very necessary to replace chemical drugs with safe alternatives from medicinal plants.

*Ocimum basilicum*, otherwise known as sweet basil, is a genus from Lamiaceae family. It is a popular herb used in traditional medicine and as a typical ingredient of the healthy Mediterranean diet. *Ocimum basilicum* contains a wide range of bioactive compounds and is a component of several food supplements, which are easily accessible as products able to maintain and promote health [12]. Moreover, basil is proved to be antioxidant, anti-inflammatory, and antihypertensive; it also acts against cancer and cardiovascular diseases [12]. These properties are in part attributed to high contents of phenolic compounds such as rosmarinic, chicoric, and caffeic acids [13]. Actually, these compounds have been demonstrated to play crucial roles in the prevention of several diseases including atherosclerosis and ischemic heart disease in addition to hepatotoxicity [14, 15].

Rosmarinic acid is known as one of the most important polyphenols [16]. It has been reported that rosmarinic acid exerts different biological activities, such as antioxidant, anticancer, anti-inflammatory, immunomodulatory, and health-enhancing activities [17–19]. Therefore, the main objective of the current study is to screen rosmarinic acid-rich extract for its toxicological effects and hepatoprotective potential in experimental animals. For that, we used a rat model of acute liver injury induced by CCl_4_ to examine the therapeutic effects of rosmarinic acid-rich extract from *Ocimum basilicum* and the mechanisms of its hepatoprotective activity.

## 2. Materials and Methods

### 2.1. Plant Material

*Ocimum basilicum* L. commonly known as sweet basil belongs to the genus *Ocimum* of the family Lamiaceae (local name: hbaq, English name: basil). It was purchased from an herbalist in Oujda city and authenticated by a botanist (Pr. A. Khalil, Department of Biology, Faculty of Sciences, Oujda, Morocco). A voucher specimen has been deposited at the Department of Biology (collection no. LO 15).

### 2.2. Preparation of Rosmarinic Acid-Rich Extract

*O. basilicum* was purchased from an herbalist in Oujda city and the rosmarinic acid-rich extract (RAE) was prepared according to a previously described procedure [20]. The extraction yielded 7.93%. The dose of the rosmarinic acid-rich extract from *O. basilicum* (200 mg/kg) was selected based on the previous efficacy studies conducted by Harnafi Hicham [21, 22] and the acute toxicity study.

### 2.3. Acute Toxicity Study

The acute oral toxicity was evaluated following the World Health Organization (WHO) guideline [23]. Briefly, adult *Albino* mice weighing 26–33 g were used to assess acute toxicity. Preseparated male and female mice were divided into seven groups (*n* = 6) according to the following protocol: the control group (group I) received only a water solution, and the group II received a 4% aqueous DMSO solution, while groups III, IV, V, VI, and VII were gavaged with the extract dissolved in 4% aqueous DMSO at doses of 2, 8, 10, 12, and 14 g/kg body weight, respectively. The animals were maintained on standard animal diet and water.

Mice treated were observed for the next 14 days. During this period for all, external general symptoms of toxicity and mortality were recorded. The LD_50_ was determined graphically from the curve: % mortality = f(extract dose).

### 2.4. Subchronic Toxicity Study

The experiment was conducted according to the protocols described by OECD Guideline 407 [24] with minimum modification. Two groups of seven mice adult male *Albino* mice weighing 26–31 g were used in this experiment. The rosmarinic acid-rich extract was administered orally on a daily basis for 28 days at a dose of 200 mg/kg, while the control group received only distilled water.

To quantify hematological and plasma biochemical parameters (liver and kidney characteristics), all animals were fasted overnight and lightly anesthetized with diethyl ether; the blood samples were then taken from their retro-orbital sinus, before and after 28 days of treatment, in tubes containing trisodium citric acid as an anticoagulant. The blood samples were immediately centrifuged (2500 rpm/15 min) to obtain plasma used for lipid analysis. Enzymatic methods using commercial kits were employed to measure the following parameters: glucose, uric acid, bilirubin, creatinine, total cholesterol, and triglyceride.

### 2.5. Hepatoprotective Effect Assay of Rosmarinic Acid-Rich Extract

#### 2.5.1. Animals

Eighteen healthy adult *Wistar* rats weighing 150–250 g were used in the study. They were bred in the Animal House of the Department of Biology (Faculty of Sciences, Oujda, Morocco) and maintained in controlled room at temperature of 22 ± 02°C with a 12 h light-dark cycle. The rats were given free access to diet and water *ad libitum*. The rats were adapted one week preceding treatment. All the experiments were performed accordance to internationally accepted standard guidelines for use of laboratory animals (approved by the local committee of use of laboratory animals, Faculty of Medicine approval number: 002016).

#### 2.5.2. Experimental Procedure

Rats were divided into three groups consisting of six animals in each group and treated for two weeks as follows: Group I served as normal control and daily received distilled water (10 mL/kg) [25]. Group II served as CCl_4_-hepatotoxicity control exposed to CCl_4_ on days seven and fourteen and was orally given distilled water. Group III rats (CCl_4_ + RAE) were exposed to CCl_4_ on days seven and fourteen, followed by the administration of rosmarinic acid-rich extract (200 mg/kg).

All rats except those in Group I received CCl_4_ intraperitoneally at a dose 1 mL/kg body weight (25% CCl_4_, solubilized in olive oil; v/v) once a week for two weeks of treatment in order to induce liver injury. Body weights of the rats were measured before and after the treatment. All animals were treated and observed daily for two weeks.

#### 2.5.3. Blood and Tissue Sample Collection

At the end of the experiment, and twelve hours after CCl_4_ injection, all of the animals were anesthetized with ethyl ether and blood samples from the abdominal aorta were collected immediately and deposited into a plastic tube containing anticoagulant solution, followed by plasma separation at 3000 rpm for 10 min and at 4°C. Samples were then kept at −20°C until the analysis of liver function parameters. Livers were removed quickly and washed in ice-cold physiological saline for the assessment of oxidative stress.

#### 2.5.4. Biochemical Analysis

All plasma parameters (alanine transaminase (ALT), aspartate transaminase (AST), alkaline phosphatase (ALP), lactate dehydrogenase (LDH), bilirubin, total protein (TP), albumin, urea, creatinine, total cholesterol, high-density lipoprotein (HDL) cholesterol, low-density lipoprotein (LDL) cholesterol, glucose, and triglyceride) were assayed enzymatically using an automated analyzer.

#### 2.5.5. Determination of Lipid Peroxides, Measured as Malondialdehyde (MDA)

In this study, the extent of lipid peroxidation in the tissues was determined by measuring the quantity of MDA, a product of membrane lipid peroxidation, according to the method described by Buege and Aust [26].

In brief, 1 g of liver from each animal was homogenized in 5 mL of PBS solution (pH = 7.4); the homogenate was then centrifuged at 14 500 rpm for 15 min in high-speed centrifuge. 1 mL of the supernatant obtained was added to 2 mL of the reagent (0.375% of thiobarbituric acid and 15% of trichloroacetic acid were dissolved in 0.25 N hydrochloric acid). Then, the mixture was heated in a boiling water bath for 30 minutes and centrifuged at 4750 rpm for 5 min. The reaction mixture was chilled, and the absorbance was measured at 535 nm.

The quantities of MDA were calculated from an extinction coefficient of 1.56 x 10^5^ M^−1^ cm^−1^. The results were expressed in nanomoles of MDA produced per milligram of tissue.

### 2.6. Statistical Analysis

Data obtained were analyzed using Student's *t*-test and one-way ANOVA. *p* values less than 0.05 were considered as statistically significant. Our results are expressed as mean ± SEM.

## 3. Results

### 3.1. Acute Oral Toxicity

We investigated the acute toxicity of the rosmarinic acid-rich extract according to the protocol described above. Indeed, the degree of acute toxicity of the RAE was assessed by determining the LD_50_.

After oral administration of RAE at doses 2, 4, 8, 10, and 12 g/kg, no mortality was observed and there were also no adverse behavioral changes throughout the observation. Thus, we note that the LD_50_ corresponding to the RAE was 14 g/kg. It is important to state that the LD_50_ of this extract was much higher than the doses with biological activities.

### 3.2. Subchronic Toxicity

Daily oral administration of RAE for 28 days did not induce any obvious symptom of toxicity in mice at a dose of 200 mg/kg body weight. No deaths or obvious clinical signs were found in test group throughout the experimental period. Normal body weight gains were observed during the study period compared to the control group.

**Figure 1:**
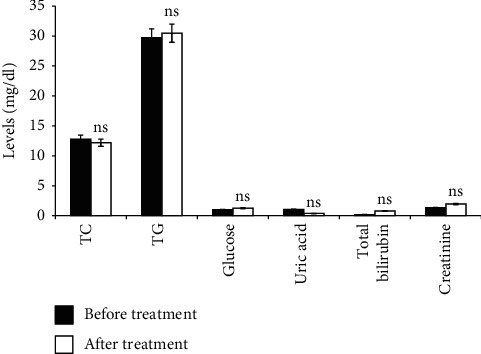
Effect of RAE on biochemical parameters tested before and after subchronic toxicity. RAE: rosmarinic acid-rich extract. Values are expressed as mean ± SEM (*n* = 7). ns: not significant.

The effects of subchronic administration of rosmarinic acid-rich extract on biochemical parameters are presented in Figure 1. According to our results, the kidney function parameters (creatinine and uric acid) did not reveal any relevant changes following administration of RAE. No statistically significant differences in the biochemical parameters (glucose, bilirubin, TG, and CT) were noted before and after the subchronic study.

### 3.3. Hepatoprotective Effect

#### 3.3.1. Effect of RAE on Body Weight Gain and the Relative Liver Weight in Rats

The effects on body weight gain and the relative liver weight in each group are shown in Table 1. Based on the results, CCl_4_ injections to the experimental animals significantly decreased rats body weight and increased relative liver weight, which was significantly prevented by the oral administration of the rosmarinic acid-rich extract.

As may be seen, intraperitoneal injection of carbon tetrachloride significantly reduced weight gain (*p* < 0.05) and increased relative liver weight (*p* < 0.01) in CCl_4_ control group comparatively to normal control group, while daily treatment with the rosmarinic acid-rich extract over the two weeks significantly improved growth performance. Indeed, treatment with the basil phenolic extract exhibited a significant decrease in the relative liver weight (*p* < 0.01) and an increase in body weight gain compared to the CCl_4_ control group.

#### 3.3.2. Effect of RAE on the Marker Enzymes Status of Liver Function

**Figure 2:**
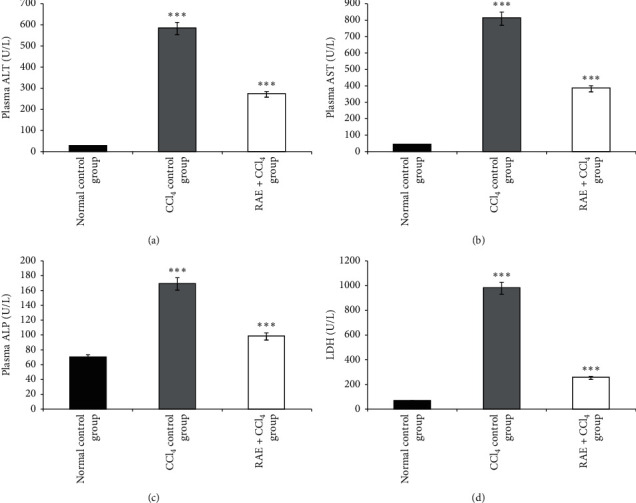
Effect of RAE on CCl_4_-induced alterations in plasma hepatic markers. RAE: rosmarinic acid-rich extract. (a) ALT: alanine aminotransferase, (b) AST: aspartate aminotransferase, (c) ALP: alkaline phosphatase, and (d) LDH: lactate dehydrogenase. Values are expressed as mean ± SEM (*n* = 6). ^*∗∗∗*^*p* < 0.001 (CCl_4_ control group versus normal control group; RAE + CCl_4_ group versus CCl_4_ control group).

The findings of the marker enzymes for liver damage illustrated in Figure 2 indicated that the intraperitoneal injection of CCl_4_ significantly (*p* < 0.001) increased the plasma levels of AST, ALT, ALP, and LDH in CCl_4_-treated group when compared with those of the control group. However, the pretreatment of RAE 200 mg/kg caused a significant decline (*p* < 0.001) in the levels of AST (−52.78%), ALT (−53.51%), ALP (−42%), and LDH (−74%), which were increased in the CCl_4_-intoxicated group.

#### 3.3.3. Effect of RAE on Plasma Direct and Total Bilirubin

**Figure 3:**
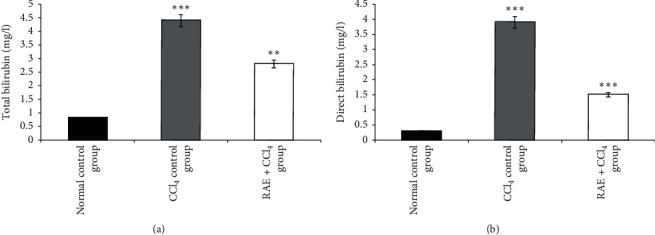
Effect of RAE on plasma total bilirubin (a) and direct bilirubin (b) in CCl_4_-intoxicated rats. RAE: rosmarinic acid-rich extract. Values are expressed as mean ± SEM (*n* = 6). ^*∗∗∗*^*p* < 0.001; ^*∗∗*^*p* < 0.01 (CCl_4_ control group versus normal control group; RAE + CCl_4_ group versus CCl_4_ control group).

Administration of CCl_4_ significantly increased the activities of direct (+1200%, *p* < 0.001) and total bilirubin levels (+450%, *p* < 0.001) when compared with the normal group. On the other hand, 200 mg/kg dose of RAE prevented CCl_4_-induced liver injury as demonstrated by significant reduction of direct (-61.5%, *p* < 0.001 and total bilirubin (−36.4%, *p* < 0.01) levels compared to CCl_4_-intoxicated group (Figure 3).

#### 3.3.4. Effect of RAE on Plasma Urea and Creatinine Levels

**Figure 4:**
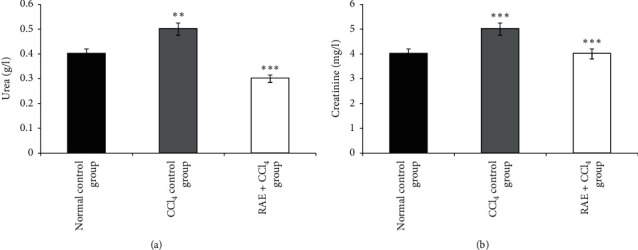
Effect of RAE on plasma urea (a) and creatinine (b) levels in CCl_4_-intoxicated rats. RAE: rosmarinic acid-rich extract. Values are expressed as mean ± SEM (*n* = 6). ^*∗∗∗*^*p* < 0.001; ^*∗∗*^*p* < 0.01 (CCl_4_ control group versus normal control group; RAE + CCl_4_ group versus CCl_4_ control group).

The results obtained are presented in Figure 4. The findings of this study indicated that the CCl_4_ treatment caused significant increases in the plasma urea (25%, *p* < 0.01) and creatinine levels (25%, *p* < 0.001) compared to the normal control group. Administration of basil phenolic extract to CCl_4_ treatment markedly (*p* < 0.001) ameliorated the induced elevation in the levels of plasma urea (−40%) and creatinine (−20%).

#### 3.3.5. Effect of RAE on Albumin and Total Protein Levels

**Figure 5:**
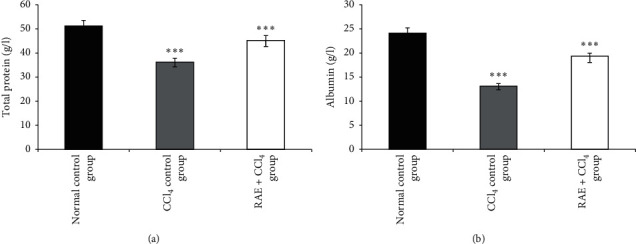
Effect of RAE on total protein (a) and albumin (b) levels in CCl_4_-intoxicated rats. RAE: rosmarinic acid-rich extract. Values are expressed as mean ± SEM (*n* = 6). ^*∗∗∗*^*p* < 0.001 (CCl_4_ control group versus normal control group; RAE + CCl_4_ group versus CCl_4_ control group).

Proteins are one of the most abundant organic molecules in living systems and have the most diverse range of functions of all macromolecules and are also the building units of the body; they regulate various physiological and metabolic processes [27]. The obtained biochemical data (Figure 5) showed that administration of CCl_4_ to rats significantly decreased the total protein content (−29.4%, *p* < 0.001) and albumin (−45.8%, *p* < 0.001) compared to the normal group. A significant increase (*p* < 0.001) in total protein (+25%) and albumin (+46%) levels was recorded in RAE-treated group compared to CCl_4_-treated group, which indicates the recovery of the protein synthesis machineries of liver.

#### 3.3.6. Effect of RAE on Lipid Profile and Glucose

**Figure 6:**
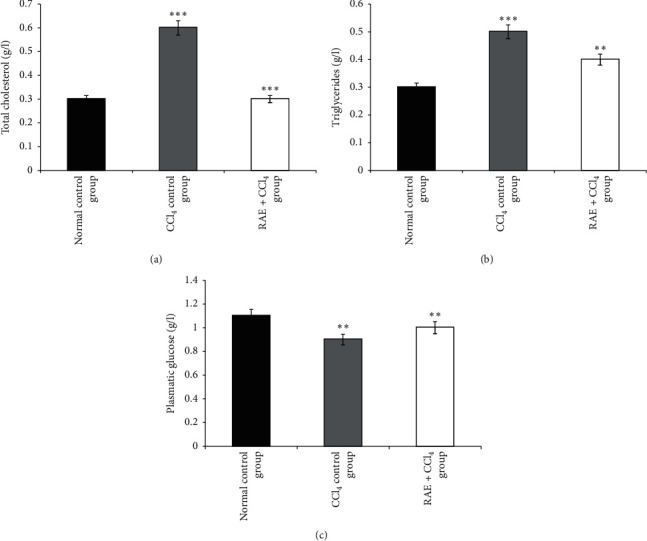
Effect of RAE on plasma total cholesterol (a), triglycerides (b), and glucose (c) in CCl_4_-intoxicated rats. RAE: rosmarinic acid-rich extract. Values are expressed as mean ± SEM (*n* = 6). ^*∗∗∗*^*p* < 0.001; ^*∗∗*^*p* < 0.01 (CCl_4_ control group versus normal control group; RAE + CCl_4_ group versus CCl_4_ control group).

**Figure 7:**
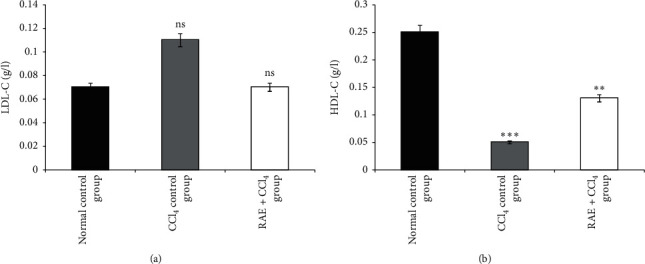
Effect of RAE on plasma lipoproteins LDL-C (a) and HDL-C (b) in CCl_4_-intoxicated rats. RAE: rosmarinic acid-rich extract. Values are expressed as mean ± SEM (*n* = 6). ^*∗∗∗*^*p* < 0.001; ^*∗∗*^*p* < 0.01; ns: not significant (CCl_4_ control group versus normal control group; RAE + CCl_4_ group versus CCl_4_ control group).

The results depicted by Figures 6 and 7 showed that, in CCl_4_-induced intoxicated rats, the plasma lipid levels (TC, TG, and LDL-C) increased (100%, 66.6%, and 57%, respectively) as compared with the control group values. Conversely, there was also a significant decline in the HDL-C level by 80% (*p* < 0.001).

However, rats pretreated with RAE remarkably exhibited a marked reversal of the plasma lipid profile (TC, TG, and LDL-C levels) compared to CCl_4_-intoxicated group. Moreover, HDL-C level significantly increased (+160%, *p* < 0.01) as compared to toxic control.

Relative to the corresponding control values, CCl_4_ injections caused a significant reduction in glucose level (*p* < 0.01). The alteration in glycaemia level was significantly recovered (*p*=0.01) after RAE administration at a dose of 200 mg/kg as compared to CCl_4_-intoxicated group (Figure 6).

#### 3.3.7. Effect of RAE on Lipid Peroxidation

**Figure 8:**
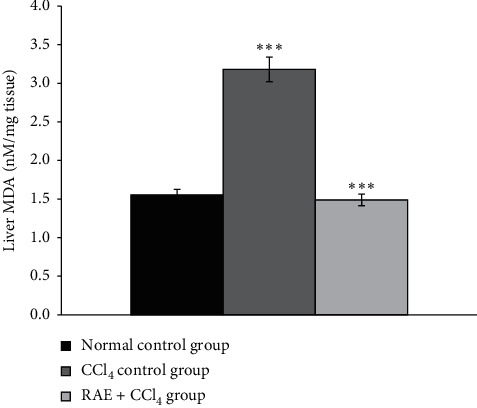
Effect of RAE on lipid peroxidation in CCl_4_-intoxicated rats. RAE: rosmarinic acid-rich extract. Values are expressed as mean ± SEM (*n* = 6). ^*∗∗∗*^*p* < 0.001 (CCl_4_ control group versus normal control group; RAE + CCl_4_ group versus CCl_4_ control group).

MDA, one of the final products of lipid peroxidation, appears to be the general marker of oxidative stress. As shown in Figure 8, the administration of CCl_4_ resulted in a significant (+105%, *p* < 0.001) increase in the lipid peroxidation marker, MDA, when compared to the control group.

Basil phenolic extract at a dose of 200 mg/kg significantly (−53%, *p* < 0.001) decreased the lipid peroxidation in RAE-treated rat liver as compared with those of CCl_4_-treated rats, indicating the hepatoprotective effect of the rosmarinic acid-rich extract.

## 4. Discussion

This study has shown that the rosmarinic acid-rich extract from *Ocimum basilicum* possesses a good safety and efficacy profile in the acute, subchronic, and hepatotoxicity study in mice and rats.

Despite the widespread use, few scientific research studies validated the safety and effectiveness of traditional remedies. The current investigation shows that the rosmarinic acid-rich extract is practically nontoxic via the oral route in mice, at least up to a maximum dose of 12 g/kg. In the acute toxicity study, it seems that the mortality and the main behavioral signs of toxicity were noted only after oral administration of relatively high dose of the phenolic extract in mice. In agreement with these findings, it may be seen that the rosmarinic acid-rich extract was assigned class 5 status (LD_50_ = 14 g/kg) and then recognized as a low toxic product, according to the chemical labelling and classification of acute systemic toxicity recommended by Organization for Economic Cooperation and Development (OECD). Since no toxic effects were observed during the acute toxicity study, further evaluation was performed to assess the subchronic toxicity of rosmarinic acid-rich extract up to 28 days in mice to provide the comprehensive toxicology data of this basil phenolic extract.

The subchronic study has long been used to assess the undesirable effects of continuous or repeated exposure of substance over a portion of the average life span of experimental animals. The 28 days' continual dose of the RAE oral toxicity study indicates that the dose of 200 mg/kg/day did not exhibit any signs of adverse effects, treatment-related signs of toxicity, or mortality. Indeed, monitoring of weight changes over the study period showed no significant differences, demonstrating no signs of toxicity. In this study, no significant changes in glucose, TG, TC, total bilirubin, and creatinine, as well as uric acid concentrations, were observed. These parameters reflect the state of carbohydrate and lipid metabolism in the liver.

The present study reports also the potential hepatoprotective activity of rosmarinic acid-rich extract from *Ocimum basilicum* against hepatic injury produced by carbon tetrachloride in rats. Hepatotoxicity induced by CCl_4_ is one of the best-characterized systems of xenobiotic-induced hepatotoxicity in experimental animals [1, 28]. Carbon tetrachloride consistently induced toxicity is characterized by the generation of reactive intermediate such as trichloromethyl radical and its derivative trichloromethyl peroxy radicals which initiate free radical-mediated lipid peroxidation leading to the accumulation of lipid-derived oxidation products that cause liver injury [29].

Generally, the extent of hepatic damage is evaluated by the increased level of cytoplasmic enzymes such as ALT, AST, ALP, and LDH; when there is hepatopathy, these marker enzymes leak into the blood. This was associated with massive centrilobular necrosis, ballooning degeneration, and cellular infiltration of the liver [30, 31].

In the preventive study experiment, CCl_4_ induced severe hepatic injury, as represented by changes in the abovementioned enzymes, besides the decreased body weight, which was consistent with previous reports [32, 33].

The increase in these biochemical enzymes may be due to tissue damage in the liver, kidneys, and heart, following changes in cell membrane permeability, or also due to increased synthesis or decreased catabolism of aminotransferases [34]. Our results revealed that the daily administration of rosmarinic acid-rich extract reversed the toxicity induced by CCl_4_ as the elevated plasma ALT, AST, ALP, and LDH activities were prevented. In this regard, we have shown that the basil phenolic extract significantly increased the abnormal plasma levels; our findings are in agreement with earlier studies [27, 35].

Urea, creatinine, albumin, and total and direct bilirubin levels were also altered by the administration of CCl_4_ which were reversed by treatment with rosmarinic acid-rich extract suggesting protection of hepatocytes from CCl_4_-mediated damage. These parameters are essential for renal function assessment and glomerular filtration [36]. On the other hand, our investigation found that this hepatotoxic agent caused a significant decline in plasma glucose level compared to normal control group.

Total protein is predominantly produced in the liver; the reduction in plasma total protein concentration associated with CCl_4_ toxicity demonstrated decrease in synthetic function of the liver. Carbon tetrachloride negatively interferes with protein metabolism probably by inhibiting the synthesis of proteins [37]. This decrease was restored towards the control value when CCl_4_-intoxicated rats treated with rosmarinic acid-rich extract.

The current results have also established that the CCl_4_ treatment could have affected the lipid metabolism of liver. CCl_4_ hepatotoxicity was characterized by significant elevation in TC, TG, and LDL-C levels and marked decrease in HDL-C in this study. Extensive accumulation of lipids is considered as a pathological condition, and when the accumulation becomes chronic, fibrotic changes occur in the cells that progress to cirrhosis and impaired liver function. The rise in cholesterol level might be due to the increased esterification of fatty acids, inhibition of fatty acids *β*-oxidation, and decreased excretion of cellular lipids [28]. However, the phenolic extract restored the lipid parameters indicating its ability to bring recovery to the liver by reducing the necrosis and fatty deposition caused by CCl_4_ toxicity.

It has been reported that lipid peroxidation, reducing activity of antioxidant enzymes, and generation of free radicals are the primary reasons of CCl_4_-induced hepatic injury [38]. Previous studies have already shown that one of the main causes of CCl_4_-induced hepatotoxicity is the generation of lipid peroxides by free radical derivatives of CCl_4_ [39]. Thus, the antioxidant activity or the inhibition of the generation of free radicals could be one of the mechanisms in the protection against CCl_4_-induced hepatotoxicity. Our findings from this study show a significant increase in the level of MDA following CCl_4_ administration indicating increased lipid peroxidation. Rosmarinic acid-rich extract at 200 mg/kg used in this study considerably normalized the abnormal elevating hepatic levels of MDA in the CCl_4_-induced hepatotoxic rats.

The HPLC profile showed that the basil phenolic extract contained four major phenolic compounds that are caftaric acid, caffeic acid, chicoric acid, and rosmarinic acid. The quantitative analysis shows that rosmarinic acid is the most abundant phenolic compound in the extract representing 87.3% [20]. On the basis of the findings obtained in the previous investigation, it can be concluded that the presence of rosmarinic acid may be the main contributing factor toward its hepatoprotective activity. All these biologically active compounds from the basil phenolic extract that showed hepatoprotective potential are likely responsible to antihepatotoxic effects as was shown in our experiments. It is possible that these multiple constituents may act synergistically, antagonistically, or additively in biological systems and the enhancing effect on repair may differ accordingly.

These compounds exhibit a variety of biological and pharmacological activities, including antioxidant activities. Therefore, it is possible that the rosmarinic acid-rich extract exerted its protective effects through the antioxidant effect or scavenging free radicals. It has been reported that there exists a high relationship between rosmarinic acid level and antioxidant activity for the plant extracts [40]. Furthermore, Domitrović et al. [41] reported that rosmarinic acid could ameliorate acute liver damage in CCl_4_-intoxicated mice, and the rosmarinic acid in the basil phenolic extract may be the factor for its hepatoprotective effect.

## 5. Conclusions

According to the results obtained, the sweet basil phenolic extract appears to be relatively nontoxic. It is interesting to note that, under the present experimental conditions, rosmarinic acid-rich extract showed hepatoprotective effects against carbon tetrachloride-induced liver damage in rats. Beneficial effect of the extract may be due to the presence of rosmarinic acid that has membrane-stabilizing effects.

## Figures and Tables

**Table 1:** Effect of RAE on the growth parameters in CCl_4_-intoxicated rats.

Groups	Weight gain (g)	Relative liver weight
Normal control group	29.83 ± 6.33	2.77 ± 0.13
CCl_4_ control group	12.00 ± 4.84^**b**^	3.83 ± 0.26^**a**^
RAE + CCl_4_ group	23.67 ± 6.9^**ns**^	2.89 ± 0.1^**a**^

RAE: rosmarinic acid-rich extract. Values are expressed as mean ± SEM (*n* = 6).^a^*p* < 0.01;^b^*p* < 0.05; ns: not significant (CCl_4_ control group versus normal control group; RAE + CCl_4_ group versus CCl_4_ control group).

## Data Availability

No data were used to support this study.
